# Sildenafil Alleviates Murine Experimental Autoimmune Encephalomyelitis by Triggering Autophagy in the Spinal Cord

**DOI:** 10.3389/fimmu.2021.671511

**Published:** 2021-05-13

**Authors:** Eduardo Duarte-Silva, Shyrlene Meiry da Rocha Araújo, Wilma Helena Oliveira, Deniele Bezerra Lós, Amanda Pires Bonfanti, Gabriela Peron, Livia de Lima Thomaz, Liana Verinaud, Christina Alves Peixoto

**Affiliations:** ^1^ Laboratory of Ultrastructure, Aggeu Magalhães Institute (IAM), Recife, Brazil; ^2^ Postgraduate Program in Biosciences and Biotechnology for Health (PPGBBS), Oswaldo Cruz Foundation (FIOCRUZ-PE)/Aggeu Magalhães Institute (IAM), Recife, Brazil; ^3^ Network of Immunity in Infection, Malignancy and Autoimmunity (NIIMA), Universal Scientific Education and Research Network (USERN), Recife, Brazil; ^4^ Postgraduate Program in Biological Sciences/Center of Biosciences, Federal University of Pernambuco (UFPE), Recife, Brazil; ^5^ Postgraduate Program in Biotechnology/Northeast Network in Biotechnology (RENORBIO), Federal University of Pernambuco (UFPE), Recife, Brazil; ^6^ Department of Structural and Functional Biology, University of Campinas (UNICAMP), Campinas, Brazil; ^7^ National Institute of Science and Technology on Neuroimmunomodulation (INCT-NIM), Oswaldo Cruz Institute, Oswaldo Cruz Foundation, Rio de Janeiro, Brazil

**Keywords:** EAE (Experimental Autoimmune Encephalomyelitis), Sildenafil citrate (Viagra), neuroinflammation, autophagy, nitrosative stress

## Abstract

Multiple Sclerosis (MS) is a neuroinflammatory and chronic Central Nervous System (CNS) disease that affects millions of people worldwide. The search for more promising drugs for the treatment of MS has led to studies on Sildenafil, a phosphodiesterase type 5 Inhibitor (PDE5I) that has been shown to possess neuroprotective effects in the Experimental Autoimmune Encephalomyelitis (EAE), an animal model of MS. We have previously shown that Sildenafil improves the clinical score of EAE mice *via* modulation of apoptotic pathways, but other signaling pathways were not previously covered. Therefore, the aim of the present study was to further investigate the effects of Sildenafil treatment on autophagy and nitrosative stress signaling pathways in EAE. 24 female C57BL/6 mice were divided into the following groups: (A) Control - received only water; (B) EAE - EAE untreated mice; (C) SILD - EAE mice treated with 25mg/kg of Sildenafil s.c. The results showed that EAE mice presented a pro-nitrosative profile characterized by high tissue nitrite levels, lowered levels of p-eNOS and high levels of iNOS. Furthermore, decreased levels of LC3, beclin-1 and ATG5, suggests impaired autophagy, and decreased levels of AMPK in the spinal cord were also detected in EAE mice. Surprisingly, treatment with Sildenafil inhibited nitrosative stress and augmented the levels of LC3, beclin-1, ATG5, p-CREB and BDNF and decreased mTOR levels, as well as augmented p-AMPK. In conclusion, we propose that Sildenafil alleviates EAE by activating autophagy *via* the eNOS-NO-AMPK-mTOR-LC3-beclin1-ATG5 and eNOS-NO-AMPK-mTOR-CREB-BDNF pathways in the spinal cord.

## Introduction

Multiple Sclerosis (MS) is a chronic neurodegenerative disease of the Central Nervous System (CNS) with a strong immune-inflammatory component underpinning is etiopathogenesis (1). Traditionally, MS has been characterized by demyelination, neuroaxonal degeneration and autoimmunity, which are the result of the activation of many transduction pathways, such as apoptosis, inflammation and excitotoxicity (2). Of note, the knowledge of these pathways is key to the development of new drugs to target MS or to repurpose the already available drugs.

Sildenafil is a Phosphodiesterase type 5 Inhibitor (PDE5I) widely used for erectile dysfunction (ED) (3), pulmonary hypertension (4) and Raynaud’s Syndrome (5). Due to its pleiotropic effects, especially regarding neuroprotection and neuroimmunomodulation, this drug has been tested in other conditions such as neurodegenerative diseases and affective disorders, such as Alzheimer’s Disease (AD) and Major Depressive Disorder (MDD) (6, 7), both of which have a strong immune-inflammatory component driving disease progression. The first study that showed the effects of Sildenafil in the Experimental Autoimmune Encephalomyelitis (EAE) model of MS was published by Pifarre et al. (8). In this study, Sildenafil at a dose of 10mg/kg was administered subcutaneously (s.c.) after disease onset and the spinal cord was analyzed. However, a previous study by our group has shown that Sildenafil at a dose of 25 mg/kg s.c. also has neuroprotective effects because it inhibits demyelination, neuroinflammation and apoptosis in the spinal cord of EAE mice, which is directly related to the motor dysfunction observed in EAE mice (9). These different studies highlighted different aspects of the same pathology and contributed to our understanding of how the disease evolves and how it could be treated, since they also highlighted different molecular targets. Furthermore, we have also demonstrated similar findings in the cuprizone model of MS (10). More recently, we demonstrated that Sildenafil at the same dose and route of administration has neuroprotective effects in the hippocampus of EAE mice, since it reduced the number of infiltrating T CD4^+^ lymphocytes, inhibited neuroinflammation and modulated synaptic plasticity and neurotransmission (11). However, other signaling pathways responsible for the mechanism of action of Sildenafil have not been explored. For instance, this is the case for autophagy, a cellular process essential to cell homeostasis and which is disrupted when there is an intense oxidative stress burden, causing the formation and accumulation of protein aggregates inside the cell. This ultimately compromises cell viability and leads to cell death (12, 13). Furthermore, autophagy is a key process in innate and adaptive immunity, in the regulation of inflammation, pathogen elimination and in MS pathogenesis (14–17). Moreover, studies have shown that autophagy is associated with neuroprotection against extra- and intracellular insults (18). Notably, in an animal model of depression induced by Chronic Unpredictable Mild Stress (CUMS), fluoxetine protected hippocampal astrocytes against stress-induced damage by promoting autophagy, which prevented cell death and contributed to the removal of damaged mitochondria (19). On the other hand, deficiency in autophagy observed in dopaminergic neurons may be a mechanism leading to increased susceptibility to cellular stress and neurodegeneration in Parkinson’s Disease (PD) (20). Therefore, the aim of the present study was to further explore the signaling pathways underlying the mechanism of action of Sildenafil in EAE, focusing on nitrosative stress and autophagy pathways.

## Materials and Methods

### Animals

Since MS is more prevalent in females than males (21), a total of 24 female C57BL/6 mice aged 8-12 weeks and weighing 25-30 g from Aggeu Magalhães Institute were used and distributed in the following experimental groups: a) CONTROL (n=8) - mice that received only vehicle (water); b) EAE (n=8) – mice submitted to EAE induction and that received only vehicle (water); c) SILD (n=8) - mice submitted to EAE induction during the daylight and that received 25 mg/kg of Sildenafil subcutaneously (s.c) during 21 days. Mice were kept under a controlled temperature (22°C) and photoperiod environment (12h/12h light/dark) and received water and standard chow *ad libitum* throughout the entire experiment. The experiment was approved by and performed in accordance with the guidelines of the Aggeu Magalhães Institute Ethics Committee/Oswaldo Cruz Foundation (87/2015 CEUA/FIOCRUZ), which is in compliance with European (EU Directive 2010/63/EU) and American (National Institutes of Health guide for the care and use of Laboratory animals) standards. Moreover, CEUA/FIOCRUZ took into consideration the principle of the three Rs (reduction, replacement, and refinement) to approve the current number of mice.

### EAE Induction

The EAE was induced as described elsewhere using naïve mice as controls (9). On the last day of the experiment, mice were anesthetized, and the spinal cord was harvested and used for the analysis of the nitrosative stress and autophagy pathways. All experiments were carried out in compliance with the ethical guidelines for animal experimentation (87/2015–CEUA/FIOCRUZ).

### Sildenafil Treatment

Sildenafil treatment (25mg/kg) (Viagra^®^, Pfizer) was administered on day post-induction 3 for a total of 21 days always during the daytime as previously described (9).

### Immunohistochemistry (IHC)

Immunohistochemistry was performed as previously described (9). Briefly, sections of all groups were incubated overnight at 4°C with the following primary antibodies: p-eNOS (Abcam, ab75639, 1:50), mTOR (Abcam, ab2732, 1:1000), Beclin-1 (Abcam, ab62557, 1:100), ATG5 (Abcam, ab228668, 1:100) and iNOS (Abcam, ab3523, 1:100. The labeling reaction was performed in six sections per group and the analysis of pixels quantification was performed in 6-8 images per group (mean value). Images of the same magnification were quantitatively analyzed using Gimp 2.6 software (GNU Image Manipulation Program, UNIX platforms). Unspecific labeling/background was removed from the quantification by using the selection and exclusion tool of the aforementioned software.

### Western Blotting (WB)

Protein extraction and Western blotting were performed as previously described elsewhere (9). Briefly, proteins (30 µg total) (n = 5 spinal cords/per group) were separated with 12% acrylamide gel. After overnight incubation with 5% non-fat milk, the membranes were incubated for four hours at room temperature with primary antibodies against p-AMPK (Cell Signaling, 2535S, 1:1000) followed by anti-rabbit HRP-conjugated antibody (ABCAM, ab6721, 1:3000; Sigma-Aldrich). For quantification, the pixel density of each immunoblot was determined using the Image J 1.38 software (http://rsbweb.nih.gov/ij/download.html; developed by Wayne Rasband, NIH, Bethesda, MD, USA). The analyses were done in duplicate and immunoblotting for β-actin (1:1,000, Sigma-Aldrich, #A2228) was performed as a loading control.

### Measurement of NO

The Griess colorimetric assay was used to measure spinal cord levels of NO by the detection of nitrite (NO_2_) resulting from oxidation of NO. Samples (n = 5 per group) were diluted fourfold with distilled water and deproteinized by adding 1/20 volume of a zinc sulfate solution (300 g/L), to give a final concentration of 15 g/L. Subsequently, centrifugation for 10 min at 3.500 g took place and 100 µL of samples were added to a 96-well ELISA in triplicate, followed by the same volume of Griess reagent. The standard curve was prepared by serial dilution of a solution of sodium nitrite (100 μM) was in PBS. After incubation for 10 min in the dark, the absorbance of the reaction product was read at 490 nm to allow information of nitrite concentration to be obtained. The absorbance of different samples was compared with the standard curve (22).

### Immunofluorescence of Paraffin-Embedded Tissue

Immunofluorescence was performed as described elsewhere (9). Briefly, samples were incubated overnight at 4°C with anti-p-CREB (Cell Signaling, #9198, 1:100), anti-BDNF (Alomone, ANT-010, 1:100) and anti-MAP1LC3A/B antibody (Biorad, AHP2167, 1:100). The labeling reaction was performed in six sections per group and the analysis of pixels quantification was performed in 6-8 images per group (mean value). Images of the same magnification were quantitatively analyzed using Gimp 2.6 software (GNU Image Manipulation Program, UNIX platforms). Unspecific labeling/background was removed from the quantification by using the selection and exclusion tool of the aforementioned software.

### Statistical Analysis

The statistical differences were analyzed by one-way ANOVA followed by Tukey’s post-test and two-way ANOVA followed by Tukey’s post-test (clinical score). The results are presented as mean ± standard deviation. All analysis was done by using Graphpad Prism (version 6.0, GraphPad Software Inc., USA) software. A p-value <0.05 indicates statistical significance.

## Results

### Sildenafil Prevents the Development of Severe Motor Dysfunction in EAE Mice

**Figure 1:**
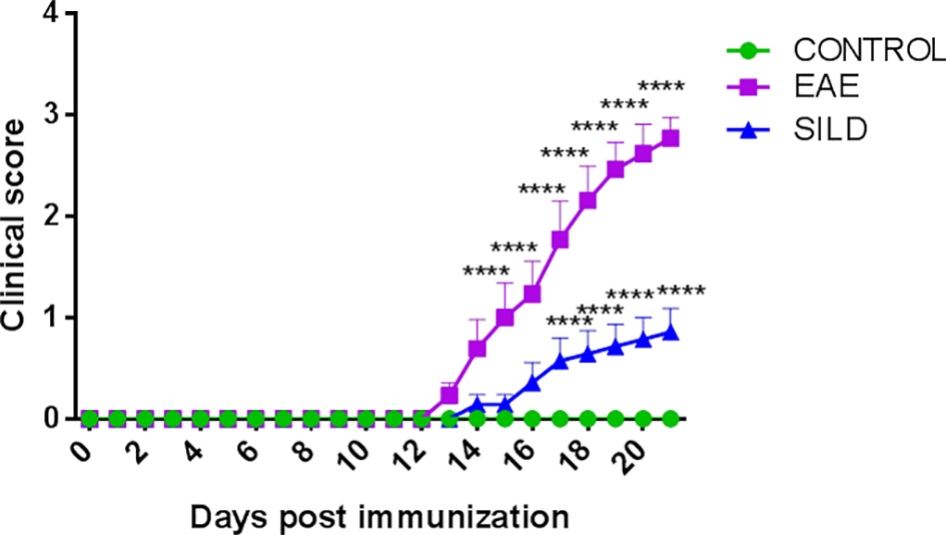
Mice clinical score. Control mice (green line) did not develop motor dysfunction, while mice with EAE (purple line) progressively developed motor dysfunction. However, the prophylactic treatment with Sildenafil (blue line) prevented the development of more severe motor symptoms. The evaluation of clinical signs of the disease was performed daily throughout the experiment and scored on a scale of 0 to 5: 0 = no sign; 1 = loss of caudal tone; 2 = weakness in hind limbs; 3 = paralysis of hind limbs; 4 = paralysis of hind limbs and weakness in anterior limbs; 5 = complete paralysis or death. The values are presented as mean ± SEM (n = 8 mice/group). Two-way ANOVA followed by Tukey’s post-test statistical analysis showed statistically significant differences between all groups over time. ****p< 0.0001. This experiment was repeated twice.

To assess whether the prophylactic administration of Sildenafil could prevent the development of motor dysfunction in EAE mice or even prevent a severe motor dysfunction, we evaluated mice daily to observe any changes in the motor function. Corroborating our previous findings, the control mice did not have any signs of motor dysfunction, which was only observed in the EAE mice. However, the prophylactic treatment with Sildenafil was able to prevent the progression of motor impairments (**Figure 1**, **Supplementary Table 1**).

### Sildenafil Decreases Nitrosative Stress Markers in the Spinal Cord of EAE Mice

Oxidative and Nitrosative Stress (O&NS) are known to be inducers of the autophagy process, as damaged molecules are constantly being generated due to cellular stress and removed by autophagy (23). However, when in excess nitrosative stress can lead to protein misfolding and aggregation, which contributes to neurodegeneration (24, 25). Taking that into consideration, drugs that have antioxidant effects can likely reduce excessive O&NS and prevent their detrimental consequences. Therefore, we subsequently established whether Sildenafil could modulate the nitrosative stress pathway using antibodies against p-eNOS, iNOS and measuring the levels of tissue nitrite $\left(NO_{2}^{-}\right)$.

**Figure 2:**
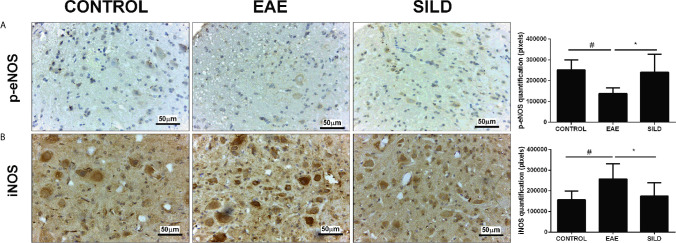
Immunohistochemistry for p-eNOS in the spinal cord **(A)**. Statistical differences were analyzed by one-way ANOVA [F (2, 15) = 6.826, P = 0.0078] followed by Tukey’s post-test. Control group presented basal expression of p-eNOS, while EAE mice had decreased labeling of this protein in immunohistochemistry). Treatment with Sildenafil increased p-eNOS immunoreactivity ^#^p < 0.05 when CONTROL vs. EAE; *p < 0.05 when EAE vs. SILD. Immunohistochemistry for iNOS in the spinal cord **(B)**. Statistical differences were analyzed by one-way ANOVA [F (2, 23) = 7.101, P = 0.0040] followed by Tukey’s post-test. Control group presented basal expression of iNOS, while EAE mice had increased labeling of this protein in immunohistochemistry. Treatment with Sildenafil decreased iNOS expression. Quantification of pixels (left panels). Values are presented as mean ± SD. ^#^p < 0.01 when CONTROL *vs.* EAE; *p < 0.05 when EAE *vs.* SILD. n= 6-8 images/group. These experiments were repeated twice.

In the p-eNOs analysis, ANOVA analysis showed a significant difference among groups (F (2, 15) = 6.826, P = 0.0078). The results showed that control mice had basal levels of p-eNOs. On the other hand, EAE mice presented decreased p-eNOS levels compared to control and sildenafil groups (p< 0.05) whereas the treatment with Sildenafil significantly augmented the p-eNOS levels compared to EAE group (p< 0.05) (**Figure 2A**). The iNOS analysis showed statistical differences among groups by one-way ANOVA (F (2, 23) = 7.101, P = 0.0040). Control mice had basal levels of iNOS. On the one hand, the EAE group had increased levels of iNOS when compared to control group (p<0.01). By its turn, treatment with Sildenafil reduced iNOS levels compared with EAE group (p< 0.05) (**Figure 2B**, **Supplementary Table 2**).

Levels of nitrite in the spinal cord showed a difference among groups by one-way ANOVA (F (2, 6) = 13.72, P = 0.0058). Control group presented basal expression of NO, while EAE mice had increased levels of nitrite compared with control group (p< 0.01). Conversely, treatment with Sildenafil reduced nitrite levels compared to EAE group (p< 0.05) (**Figure 4C**).

### Sildenafil Modulates Autophagy Markers to Improve EAE

The link between autophagy dysfunction and neurodegenerative diseases is well established. In many diseases, such as Parkinson’s Disease (PD) and Alzheimer’s Disease (AD), protein aggregation occurs due to the inability of the cellular machinery to remove them and this usually is accompanied by neurotoxicity and neurodegeneration (26). In the case of MS, it was experimentally demonstrated that protein aggregation contributes to neurodegeneration (12). In this regard, drugs that can target this pathway can likely be strong candidates to treat such diseases because they can likely remove protein aggregates and reestablish cellular proteostasis. To determine whether Sildenafil could modulate autophagy pathway, we used antibodies against autophagy and autophagy-related molecules, such as LC3, beclin-1, ATG5, p-CREB, BDNF and mTOR.

**Figure 3:**
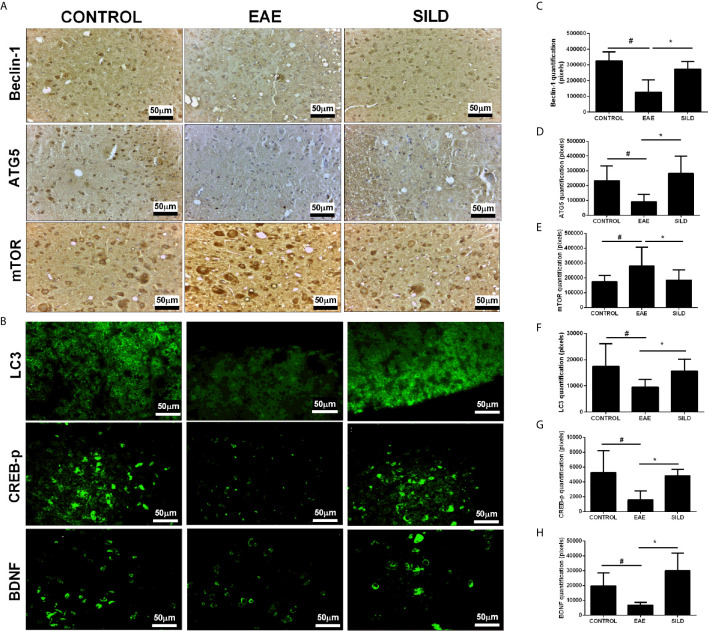
**(A)** Immunohistochemistry for beclin-1 in the spinal cord. Statistical differences were analyzed by one-way ANOVA [F (2, 9) = 11.05, P = 0.0038] followed by Tukey’s post-test. Control group presented basal expression of beclin-1, while EAE mice had decreased labeling of this protein in immunohistochemistry. Treatment with Sildenafil increased beclin-1 immunoreactivity. Immunohistochemistry for ATG5 in the spinal cord. Statistical differences were analyzed by one-way ANOVA [F (2, 14) = 7.343, P = 0.0066] followed by Tukey’s post-test. Control group presented basal expression of ATG5, while EAE mice had decreased labeling of this protein in immunohistochemistry. Treatment with Sildenafil increased ATG5 immunoreactivity. Immunohistochemistry for mTOR in the spinal cord. Statistical differences were analyzed by one-way ANOVA [F (2, 30) = 4.817, P = 0.0153] followed by Tukey’s post-test. Control group presented basal expression of mTOR while EAE mice had increased labeling of this protein in immunohistochemistry. Treatment with Sildenafil decreased immunoreactivity mTOR immunoreactivity. **(C–E)** Quantification of pixels. Values are presented as mean ± SD. ^#^p < 0.05 when CONTROL *vs*. EAE; *p < 0.01 when EAE *vs.* SILD. n= 6-8 images/group. These experiments were repeated twice. **(B)** Immunohistochemistry for LC3 in the spinal cord. Statistical differences were analyzed by one-way ANOVA [F (2, 31) = 8.718, P = 0.0010] followed by Tukey’s post-test. Control group presented basal expression of LC3, while EAE mice had decreased labeling of this protein in immunohistochemistry. Treatment with Sildenafil increased LC3 immunoreactivity. ^#^p < 0.05 when CONTROL *vs*. EAE; *p < 0.01 when EAE *vs.* SILD. Immunohistochemistry for p-CREB in the spinal cord. Statistical differences were analyzed by one-way ANOVA [F (2, 10) = 6.987, P = 0.0126] followed by Tukey’s post-test. Control group presented basal expression of p-CREB, while EAE mice had decreased labeling of this protein. Treatment with Sildenafil increased p-CREB immunoreactivity. ^#^p < 0.05 when CONTROL *vs*. EAE; *p < 0.01 when EAE *vs.* SILD. Immunohistochemistry for BDNF in the spinal cord. Statistical differences were analyzed by one-way ANOVA [F (2, 15) = 14.26, P = 0.0003] followed by Tukey’s post-test. Control group presented basal expression of BDNF, while EAE mice had decreased labeling of this protein. Treatment with Sildenafil increased BDNF immunoreactivity. ^#^p < 0.05 when CONTROL *vs*. EAE; *p < 0.001 when EAE *vs.* SILD **(F–H)** Quantification of pixels. Values are presented as mean ± SD. n= 6-8 images/group. These experiments were repeated twice.

Notably, we investigated whether Sildenafil could target the nucleation and elongation phase of autophagy characterized by the participation of LC3-beclin-1 and ATG5, respectively (20, 27). ANOVA analysis showed a significant difference among groups in LC3(F (2, 31) = 8.718, P = 0.0010). EAE mice, on the one hand, had decreased levels of LC3 in comparison to control group (p< 0.05). On the other hand, treatment with Sildenafil increased LC3 levels when compared to untreated mice (p<0.01) (**Figures 3B, F**). In the beclin-1 analysis, ANOVA analysis showed a significant difference among groups (F (2, 9) = 11.05, P = 0.0038). Untreated mice had decreased expression of beclin-1 when compared to control mice (p<0.01), which was reversed by treatment with Sildenafil (p< 0.05) (**Figures 3A, C**). ANOVA analysis showed a significant difference among groups in ATG5 analysis (F (2, 14) = 7.343, P = 0.0066). On the one hand, untreated mice had diminished levels of ATG5 when compared to control group (p< 0.05). On the other hand, Sildenafil augmented ATG5 levels when compared to the EAE group (p<0.01) (**Figures 3A, D**). ANOVA analysis showed a significant difference among groups in mTOR (F (2, 30) = 4.817, P = 0.0153). EAE group had increased expression of mTOR in comparison to the control group (p< 0.05), while mice that received Sildenafil had mTOR expression reduced (p< 0.05) (**Figures 3A, E**, **Supplementary Table 2**).

Regarding p-CREB, ANOVA analysis showed a significant difference among groups [F (2, 10) = 6.987, P = 0.0126]. Untreated mice displayed reduced p-CREB levels in comparison to the control mice (p< 0.05), while treatment with Sildenafil reversed this reduction (p< 0.05) (**Figures 3B, G**). Finally, ANOVA analysis showed a significant difference among groups in BDNF [F (2, 15) = 14.26, P = 0.0003]. Untreated mice had lower levels of BDNF when compared to control group (p< 0.05). However, treatment with Sildenafil increased BDNF levels in comparison to the EAE group (p< 0.001) (**Figures 3B, H**, **Supplementary Table 2**).

### Sildenafil Modulates AMPK to Improve EAE

**Figure 4:**
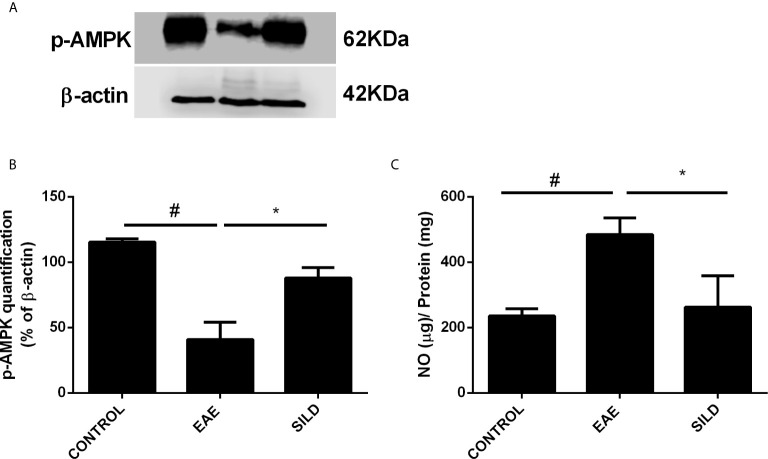
Western blot for p-AMPK **(A)** Statistical differences were analyzed by one-way ANOVA [F (2, 3) = 34.57, P = 0.0085] followed by Tukey’s post-test. Control group presented basal expression of p-AMPK, while EAE mice displayed decreased p-AMPK levels. Treatment with Sildenafil increased p-AMPK levels. **(B)** Densitometric analysis of p-AMPK. Values are presented as mean ± SD. ^#^p < 0.01 when CONTROL *vs*. EAE; *p <0.05 when EAE *vs.* SILD n= 5 mice/group. This experiment was repeated twice. **(C)** Levels of nitrite in the spinal cord showed difference among groups by one-way ANOVA [F (2, 6) = 13.72, P = 0.0058]. Control group presented basal expression of NO, while EAE mice had increased levels of nitrite compared with control group. Conversely, treatment with Sildenafil reduced nitrite levels compared to EAE group. Values are presented as mean ± SD. ^#^p < 0.01 when CONTROL *vs*. EAE; *p < 0.05 when EAE *vs.* SILD. n= 5 mice/group. This experiment was repeated twice.

AMP-activated protein kinase (AMPK) is not only an energy balance regulator, but it also plays a key role in the modulation of autophagy in an mTOR-dependent and independent fashion, such as *via* the modulation of the transcription factor Forkhead box O3 (FOXO3), known to induce the expression of autophagy genes, such as the ones that codify for beclin-1 and ATG5 (28–30). In this regard, drugs that can modulate AMPK may be of relevance to achieve autophagy induction in neurodegenerative diseases. To establish whether Sildenafil could modulate the AMPK and thus indirectly modulate autophagy, we next used an antibody against p-AMPK. ANOVA analysis showed a significant difference among groups (F (2, 3) = 34.57, P = 0.0085). The results showed that control mice had basal levels of p-AMPK. Untreated mice, on the one hand, presented decreased levels of p-AMPK when compared to control mice (p<0.01). Nonetheless, treatment with Sildenafil increased the levels of p-AMPK in comparison to EAE mice (p< 0.05) (**Figures 4A, B**).

## Discussion

In this study, we further investigated the molecular pathways underpinning the improvement observed in EAE mice treated with Sildenafil. Previously, we have demonstrated that Sildenafil decreases the clinical score of EAE mice by modulation of apoptotic pathways (9). Here, we corroborated this finding and demonstrated that this amelioration is also caused by the modulation of other signaling pathways, such as the nitrosative stress and autophagy pathways.

Dysfunction in the cellular redox status plays an important role in the pathogenesis of many diseases, including, but not limited to MS, Major Depressive Disorder (MDD) and Chagas disease (31–33). Increased levels of Reactive Oxygen Species (ROS) as well as Reactive Nitrogen Species (RNS), such as Nitric Oxide (NO) and nitrite can be detrimental. ROS and RNS can react with proteins, lipids, and nucleic acids, causing damage to these molecules. Furthermore, by damaging the aforementioned molecules, ROS and RNS can generate neoepitopes against which an immune response can be mounted (31). Inducible Nitric Oxide Synthase (iNOS) is usually activated during inflammation and leads to high production of NO, within the micromolar range. It is noteworthy that NO can also be a source of RNS, which can damage the cellular constituents. Nitration of proteins is considered to be a mechanism driving disease progression in AD, PD and Lateral Amyotrophic Sclerosis (ALS) (34, 35). However, not much is known about it in the context of MS. NO can react with the cysteine residue of proteins by S-nitrosylation (SNO) and thereby affect protein structure and function, leading to their activation, deactivation or even aggregation, which is toxic to cells and drives neurodegeneration (36). Of key importance is when SNO modification occurs in pathways responsible for *proteostasis* and *cytoprotection*, such as the antioxidant and anti-apoptotic pathways. As a consequence, dysfunctional redox states and impaired autophagy facilitate protein aggregation and its permanence in the cell, affecting cell viability. In this regard, high amounts of NO facilitate the formation of protein aggregates (36, 37).

On the other hand, Endothelial Nitric Oxide Synthase (e-NOS) is responsible for basal NO production (nanomolar range), which regulates, for instance, vascular tone (38, 39). NO takes part in the NO-soluble guanylate cyclase (sGC)-Cyclic guanosine monophosphate (cGMP)-dependent protein kinase (PKG) pathway. Of note, accumulation of cGMP exerts anti-inflammatory effects by reducing the levels of Intercellular Adhesion Molecule 1 (ICAM) and Vascular Cellular Adhesion Molecule (VCAM) in the cerebellum, which inhibits the traffic of leukocytes to the CNS (40). Furthermore, NO can also activate AMPK and reduce inflammation (41). Interestingly, AMPK is activated in response to IL-10 and favors macrophage polarization towards the anti-inflammatory or M2 phenotype (42). This is corroborated by our previous study (11) and this suggests that the switching of microglia to the M2 phenotype is probably due to the increased levels of IL-10 and p-AMPK in mice treated with Sildenafil. Furthermore, we showed that EAE is characterized by lowered expression of p-eNOS, but high levels of iNOS and NO, which favors nitrosative stress and protein aggregation. However, treatment with Sildenafil decreased iNOS and NO levels, while increasing the expression of eNOS.

It is worthy to mention that activated AMPK phosphorylates and activates eNOS, leading to NO production, which in turn activates AMPK (41, 43). Data have shown that oxidative and nitrosative stress (O&NS) pathways play a key role in EAE and MS, characterized by high levels of oxidative stress markers as well as lowered levels of antioxidants (44–46). In a second vein, data have shown that Sildenafil is able to inhibit ROS and elevate the expression of antioxidant molecules, such as Superoxide Dismutase (SOD) and catalase (47). A previous study by our laboratory conducted with the cuprizone model of MS showed that Sildenafil led to increased levels of eNOS and p-AMPK (22). All in all, these data show that Sildenafil, when administered before disease symptoms, inhibits nitrosative stress and improves EAE. Furthermore, by stimulating eNOS, Sildenafil triggers the activation of the NO-sCG-cGMP-PKG pathway, which leads to the activation of AMPK.

AMPK is a protein known to be a regulator of energy balance. Interestingly, AMPK also regulates autophagy (48). Activated AMPK phosphorylates Tuberous Sclerosis 1 and 2 (TSC1/2) and inhibits mTORC1 complex, thus promoting autophagy (49). Furthermore, AMPK activation leads to inhibition of Nuclear Factor kappa B (NFκB) (50, 51). Moreover, activated AMPK also inhibits iNOS and thus inflammation (52). A study conducted in our laboratory has shown that Sildenafil increased the expression of p-AMPK in the cuprizone model of MS (22). In this regard, here we showed that EAE mice treated with Sildenafil presented increased levels of p-AMPK, which further contributes to inhibition of neuroinflammation in the spinal cord. Interestingly, AMPK activated by NO phosphorylates IκB kinase (IKK) and prevents NFκB activation, thus exerting an anti-inflammatory effect (41). Furthermore, activated AMPK can indirectly inhibit NFκB by activating Sirtuin 1 (SIRT1), FOXO and Peroxisome Proliferator-Activated Receptor-Gamma Coactivator-1α (PGC1α) (49). In sum, our results suggest that activation of AMPK inhibits neuroinflammation and takes part in mTOR inhibition and autophagy induction. In fact, we reported here that Sildenafil increases p-AMPK and LC3 levels, while decreases mTOR levels, which likely suggests that Sildenafil favors and promotes autophagy in the spinal cord of EAE mice and thus exerts a neuroprotective effect.

Lower LC3-I/II and beclin-1 expression with consequent impaired autophagy were shown to occur in the spinal cord of EAE mice (53, 54) and induction of autophagy *via* the Cannabinoid Receptor 2 (CBR2) ameliorated EAE (55). Furthermore, it is reported that the expression of ATG5 is elevated in EAE (56), which is not corroborated by our findings and can likely be explained by differences in the analyzed samples and different stages of disease (purified T cells from EAE mice and postmortem brain tissue *versus* spinal cord as presented in this study). Here, we show that untreated mice had reduced levels of LC3, beclin-1 and ATG5 while showing an increase in mTOR expression. However, Sildenafil administration led to elevated expression of LC3, beclin-1 and ATG5, while displaying reduced mTOR levels. Furthermore, we previously reported increased IL-10 levels followed by Sildenafil treatment (11), which can further suppress the activity of mTOR and increase autophagy and mitophagy (57). Although the functional consequences of the activation of the autophagy pathways were not investigated here, one could reasonably argue that this process may lead to the removal of protein aggregates previously reported to occur during EAE and which are responsible for neuronal death by apoptosis (12, 13). Accordingly, we have previously demonstrated that during EAE the intrinsic and extrinsic pathways of apoptosis, as well as other signaling pathways that control cell survival/death, are activated, causing neuron death, which was rescued after Sildenafil treatment (9). Altogether, our results suggest that Sildenafil promotes autophagy and exerts neuroprotective effects by increasing IL-10 and AMPK levels and by modulating the expression of LC3, beclin-1 and ATG5 in the spinal cord of EAE mice.

The cAMP-response Element Binding Protein (CREB) is a transcription factor that binds to cAMP Response Element (CRE) and promotes transcription of target genes, including Brain Derived Neurotrophic Factor (BDNF), a neurotrophin that mediates neuroplasticity in the central and peripheral nervous system. Via CREB-BDNF pathway, neurons undergo neurogenesis, differentiation, survival, neurite outgrowth and synaptogenesis (58, 59). Interestingly, mTOR inhibition by rapamycin was accompanied by phosphorylation of CREB, which protected against neurodegeneration induced by amyloid-β (60). Furthermore, it is known that CREB can activate autophagy genes *via* the Farnesoid X receptor (FXR)/CREB signaling pathway whereby FXR inhibits CREB activation (61–63). By its turn, AMPK inhibits FXR and thus increases CREB activation (64, 65). Regarding BDNF, it was shown that increased levels of BDNF were associated with inhibition of autophagy (66). However, other studies have shown that BDNF can also promote autophagy (67). In diabetic rats, BDNF-TrkB pathway exerted antidepressant effect of hydrogen sulfide (H_2_S) by triggering autophagy in the hippocampus (68). In another study with cortical neurons submitted to oxygen deprivation *in vitro*, BDNF exerted neuroprotective effects by increasing autophagy *via* the PI3K/Akt/mTOR/p70S6K pathway. Interestingly, BDNF administration led to decreased mTOR levels (69). Therefore, we postulate that the increased levels of CREB/BDNF may induce autophagy and ameliorate disease pathology and may also be involved in the reduction of mTOR expression, which could further trigger autophagy. However, future studies are needed to corroborate this hypothesis.

**Figure 5:**
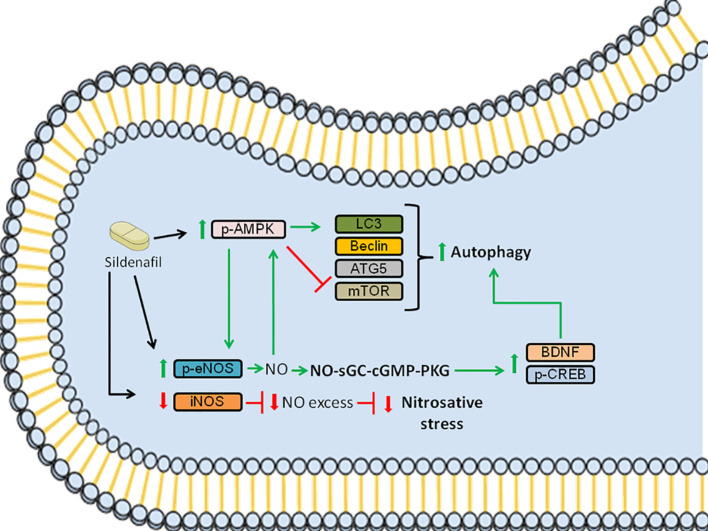
Schematic summarizing the signaling pathways modulated by Sildenafil to improve EAE pathology. Sildenafil increases the levels of p-AMPK and reduces the levels of mTOR, which leads to an increase in the levels of LC3, promoting autophagy. Furthermore, Sildenafil enhances the expression of p-eNOS, leading to the production of NO, which further activates AMPK. Moreover, NO triggers the activation of the NO-sGC-cGMP-PKG pathway, which leads to increased levels of p-CREB and BDNF, further promoting autophagy. Furthermore, Sildenafil inhibits iNOS, which when activated produces NO in excess, thus inhibiting nitrosative stress. Altogether, the activation of the aforementioned signaling pathways improves EAE pathology. Red bars: inhibition; Green arrows: activation.

In summary, our data showed that Sildenafil ameliorates EAE by activating signaling pathways that mitigate nitrosative stress and by triggering autophagy pathway (**Figure 5**). However, the use of the subcutaneous route of administration has a clear translational limitation since Sildenafil is mainly taken *via* the oral route. Furthermore, despite the fact that PDE5Is are promising drugs to treat neurodegenerative disorders, Sildenafil is currently only prescribed to MS patients to treat ED (7, 38, 70–76). Moreover, only few clinical trials were performed to elucidate the efficacy and effects of these drugs. Notably, they have shown that treatment with PDE5Is led to enhanced cognitive performance and function (77–79) and improved overall life quality of the MS patients, which included life as a whole, family life and social contacts (80). However, the lack of clinical data makes it hard to ensure that MS patients taking these drugs would have a better prognosis. Therefore, future experimental studies, as well as clinical trials with MS patients, need to be performed to provide more insights into disease pathogenesis and disease progression. This would allow a more in-depth view of PDE5Is mechanism of action and how it could help in the treatment of neurodegenerative diseases, such as MS. In conclusion, we demonstrate that Sildenafil alleviates EAE *via* the eNOS-NO-AMPK-mTOR-LC3-beclin1-ATG5 and eNOS-NO-AMPK-mTOR-CREB-BDNF pathways in the spinal cord, which triggers autophagy and consequently improves disease.

## Data Availability Statement

The original contributions presented in the study are included in the article/**Supplementary Material**. Further inquiries can be directed to the corresponding authors.

## Ethics Statement

The animal study was reviewed and approved by Aggeu Magalhães Institute Ethics Committee/Oswaldo Cruz Foundation (87/2015 CEUA/FIOCRUZ).

## Author Contributions

CP conceived and supervised this project. ED-S performed the experiment, literature search, data collection, data analysis, wrote the manuscript, and created the figures. SR, WO, DL, AB, GP, and LL provided help during the experiment and with sample processing. LV critically revised the manuscript. All authors approved the submitted version.

## Funding

The authors express their gratitude to Oswaldo Cruz Foundation of Pernambuco (FIOCRUZ-PE), Research Excellence Program - Aggeu Magalhães Institute (IAM PROEP#400208/2019-9), Knowledge Generation Program – Oswaldo Cruz Foundation (FIOCRUZ; #VPPCB-007-FIO-18-2-17), The Brazilian National Institute of Science and Technology on Neuroimmunomodulation (INCT-NIM; #465489/2014-1), and Brazilian National Council for Scientific and Technological Development (CNPq; #301777/2012-8) for research support. This study was funded in part by the Coordenação de Aperfeiçoamento de Pessoal de Nível Superior - Brasil (CAPES) - Finance Code 001. The funders had no role in study design, data collection and analysis, decision to publish, or preparation of the manuscript.

## Conflict of Interest

The authors declare that the research was conducted in the absence of any commercial or financial relationships that could be construed as a potential conflict of interest.
