# Supplementary material for: Sildenafil Alleviates Murine Experimental Autoimmune Encephalomyelitis by Triggering Autophagy in the Spinal Cord
Source: Front Immunol. 2021 May 13;12:671511. doi: 10.3389/fimmu.2021.671511 (PMC8156813; doi:10.3389/fimmu.2021.671511)
Supplement: Supplementary file 1 [file Table_1.doc]

Table 1. Statistical results obtained from two-way ANOVA (clinical score).

| **two-way ANOVA** | | | | | |  |
| --- | --- | --- | --- | --- | --- | --- |
| **Clinical score** |  | SS | DF | MS | F | P |
|  | Interaction | 124,4 | 42 | 2,962 | 19,87 | < 0,0001 |
|  | Row Factor | 166,1 | 21 | 7,910 | 53,06 | < 0,0001 |
|  | Column Factor | 55,58 | 2 | 27,79 | 186,4 | < 0,0001 |
|  | Residual | 68,88 | 462 | 0,1491 | 19,87 | < 0,0001 |
