# Supplementary material for: Sildenafil Alleviates Murine Experimental Autoimmune Encephalomyelitis by Triggering Autophagy in the Spinal Cord
Source: Front Immunol. 2021 May 13;12:671511. doi: 10.3389/fimmu.2021.671511 (PMC8156813; doi:10.3389/fimmu.2021.671511)
Supplement: Supplementary file 2 [file Table_2.doc]

Table 2. Statistical results obtained from one-way ANOVA.

| **ANOVA** | | | | | | |
| --- | --- | --- | --- | --- | --- | --- |
| **eNOS** |  | SS | DF | MS | F | P |
|  | Treatments | 4.75e+10 | 2 | 2.375e+10 | 6.828 | 0.0078 |
|  | Residuals | 5.22e+10 | 15 | 3.48e+9 | - | - |
|  | Total | 9.97e+10 | 17 | - | - | - |
|  |  | SS | DF | MS | F | P |
| **iNOS** | Treatments | 5,325e+010 | 2 | 2,662e+010 | 7,101 | 0,0040 |
|  | Residuals | 8,623e+010 | 23 | 3,749e+009 | - | - |
|  | Total | 1,395e+011 | 25 | - | - | - |
|  | | SS | DF | MS | F | P |
| **Beclin-1** | Treatments | 9,164e+010 | 2 | 4,582e+010 | 11,05 | 0,0038 |
|  | Residuals | 3,731e+010 | 9 | 4,146e+009 | - | - |
|  | Total | 1,290e+011 | 11 | - | - | - |
|  |  | SS | DF | MS | F | P |
| **ATG5** | Treatments | 1,117e+011 | 2 | 5,585e+010 | 7,343 | 0,0066 |
|  | Residuals | 1,065e+011 | 14 | 7,606e+009 | - | - |
|  | Total | 2,182e+011 | 16 | - | - | - |
|  |  | SS | DF | MS | F | P |
| **mTOR** | Treatments | 7,376e+010 | 2 | 3,688e+010 | 4,817 | 0,0153 |
|  | Residuals | 2,297e+011 | 30 | 7,656e+009 | - | - |
|  | Total | 3,034e+011 | 32 | - | - | - |
|  |  | SS | DF | MS | F | P |
| **LC3** | Treatments | 3,440e+008 | 2 | 1,720e+008 | 8,718 | 0,0010 |
|  | Residuals | 6,115e+008 | 31 | 1,973e+007 | - | - |
|  | Total | 9,555e+008 | 33 | - | - | - |
|  |  | SS | DF | MS | F | P |
| **CREB-p** | Treatments | 3,889e+007 | 2 | 1,944e+007 | 6,987 | 0,0126 |
|  | Residuals | 2,783e+007 | 10 | 2,783e+006 | - | - |
|  | Total | 6,672e+007 | 12 | - | - | - |

**(Table 1 continued on the next page)**

|  |  | SS | DF | MS | F | P |
| --- | --- | --- | --- | --- | --- | --- |
| **BDNF** | Treatments | 1,715e+009 | 2 | 8,573e+008 | 14,26 | 0,0003 |
|  | Residuals | 9,016e+008 | 15 | 6,010e+007 | - | - |
|  | Total | 2,616e+009 | 17 | - | - | - |
|  |  | SS | DF | MS | F | P |
| **NO** | Treatments | 112155 | 2 | 56078 | 13,72 | 0,0058 |
|  | Residuals | 24522 | 6 | 4087 | - | - |
|  | Total | 136677 | 8 | - | - | - |
|  |  | SS | DF | MS | F | P |
| **p-AMPK** | Treatments | 5669 | 2 | 2834 | 34,57 | 0,0085 |
|  | Residuals | 246,0 | 3 | 82,00 | - | - |
|  | Total | 5915 | 5 | - | - | - |
